# KRAS mutations as essential promoters of lymphangiogenesis via extracellular vesicles in pancreatic cancer

**DOI:** 10.1172/JCI161454

**Published:** 2022-07-15

**Authors:** Radu Pirlog, George A. Calin

**Affiliations:** 1 Research Center for Functional Genomics Biomedicine and Translational Medicine, Iuliu Hatieganu University of Medicine and Pharmacy, Cluj-Napoca, Romania.; 2 Department of Translational Molecular Pathology, Division of Pathology, and; 3 Center for RNA Interference and Non-Coding RNAs, The University of Texas MD Anderson Cancer Center, Houston, Texas, USA.

## Abstract

Kirsten rat sarcoma virus (*KRAS*) gene mutations are present in more than 90% of pancreatic ductal adenocarcinomas (PDACs). KRAS^G12D^ is the most frequent alteration, promoting preneoplastic lesions and associating with a more aggressive phenotype. These tumors possess increased intratumoral lymphatic networks and frequent lymph node (LN) metastases. In this issue of the *JCI*, Luo, Li, et al. explored the relationship between the presence of the KRAS^G12D^ mutation and lymphangiogenesis in PDAC. The authors used in vitro and in vivo models and an elegant mechanistic approach to describe an alternative pathway for lymphangiogenesis promotion. KRAS^G12D^ induced SUMOylation of heterogenous nuclear ribonucleoprotein A1 (hnRNPA1) via SAE1 and SUMO2 activation. SUMOylated hnRNPA1 was loaded into extracellular vesicles (EVs) and internalized by human endothelial lymphatic cells (HLEC). Further, SUMOylated hnRNPA1 promoted lymphangiogenesis and LN metastasis by stabilizing prospero homeodomain protein 1 (*PROX1*) mRNA. These data provide mechanistic insight into cancer lymphangiogenesis with the potential for developing biomarkers and RAS pathway therapeutics.

## KRAS mutations in pancreatic cancers

Pancreatic cancer is the deadliest gastrointestinal malignancy, as the fourth leading cause of cancer-related death and with one of the lowest 5-year survival rates, at only 11% (1). Pancreatic cancer is most often detected in advanced stages — when few therapeutic options are available — due to the lack of screening methods and paucity of symptoms in early phases. The main histologic type is pancreatic ductal adenocarcinoma (PDAC), which carries *KRAS* gene mutations as molecular signatures in more than 90% of cases (2). As a driver gene in pancreatic cancer, *KRAS* alteration promotes the development of epithelial dysplasia, pancreatic intraepithelial neoplasias (PanINs), and pancreatic mucinous neoplasms (IPMNs) (3). Until recently, *KRAS* mutations were known as undruggable targets due to the structural difficulty of designing specific inhibitors (4). In pancreatic cancer, *KRAS^G12D^* is the most common *KRAS* mutation, inducing an aggressive phenotype via the activation of MAPK, PI3K, and Ras-like GEF (RalGEF) pathways (3, 5). Pancreatic tumors harboring the *KRAS^G12D^* mutation show a particular disposition — with tumor cells surrounding lymphatic vessels, lymphatic vessel remodeling, and increased lymphangiogenesis — which facilitates intratumoral lymphatic vessel invasion and LN spreading (6). Consequently, two-thirds of patients with PDAC present with LN metastases at the time of diagnosis (7).

## KRAS^G12D^-driven SUMOylation of hnRNPA1

A more in-depth understanding of the genomic landscape of cancer using translational approaches can provide insights into the genetic alterations and unravel oncogenic pathways and possible therapeutic targets (8). In this issue of the *JCI*, Luo, Li, et al. carefully investigated the molecular mechanism behind KRAS^G12D^-induced lymphangiogenesis in pancreatic cancer. The authors used a large spectrum of in vitro and in vivo models to analyze intercellular communication between malignant cells and human endothelial lymphatic cells (HLEC) by characterizing the EVs and intracellular pathways (9). Based on the group’s previous results, which show that lymphangiogenesis is promoted by cancer-related genes interacting with RNA binding proteins, the authors investigated the role of heterogenous nuclear ribonucleoprotein A1 (hnRNPA1) in promoting lymphangiogenesis in PDAC. Notably, hnRNPA1 is overexpressed in PDAC, with expression levels correlating with the *KRAS^G12D^* mutation (10).

Analysis of the hnRNPA1 expression in serum samples from patients with PDAC *KRAS^G12D^* mutations collected from two independent clinical centers showed that elevated hnRNPA1 was associated with reduced overall and disease-free survival. In *KRAS^G12D^* PDAC, hnRNPA1 is overexpressed in EVs and LN metastases, suggesting that hnRNPA1 promotes LN metastasis via intercellular shuttling. The influence of hnRNPA1 on promoting PDAC metastasis was highlighted by Luo, Li, et al. using a popliteal lymphatic metastasis mice model (9). EVs high in hnRNPA1 acted directly on lymphatic endothelial cells to increase lymphangiogenesis and LN metastasis. Interestingly, these effects on lymphangiogenesis were observed only using the PANC-1 cell line, which harbors the *KRAS^G12D^* mutation (9). These results suggest that the KRAS^G12D^ protein has an active role in upregulating hnRNPA1 protein expression.

Additional investigation into the molecular mechanism by which KRAS^G12D^ upregulates hnRNPA1 revealed a structural difference between cytosolic hnRNPA1 and the hnRNPA1 loaded in EVs. When isolated from PDAC EVs, hnRNPA1 had a higher molecular weight than the intracellular form, suggesting the presence of additional structural post-translational modifications (PTMs). The authors screened for PTMs involved in hnRNPA1 loading into EVs and found that a SUMOylation modifier, SUMO2, was directly bound to hnRNPA1, enhancing its packaging in PDAC EVs (9).

**Figure 1:**
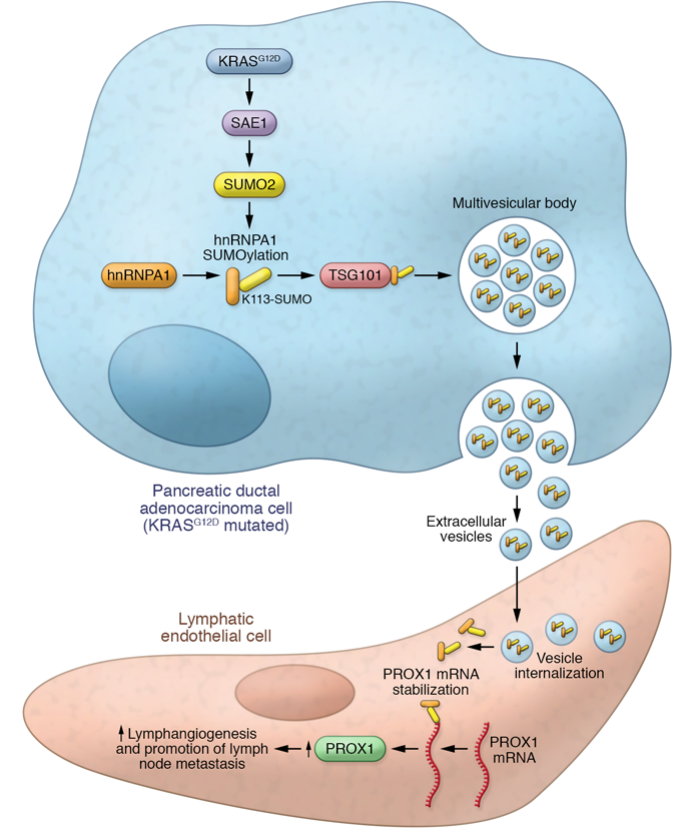
The proposed mechanism of KRASG12D-induced lymphangiogenesis in PDAC. PDAC cells with the KRAS^G12D^ mutation upregulate SAE1, which triggers SUMOylation. Subsequent SUMOylation of hnRNPA1 at lysine 113 enables interaction with a component of the endosomal sorting complex, TSG101. EVs are loaded with SUMOylated hnRNPA1 and released into the tumor microenvironment. Local lymphatic endothelial cells internalizing EVs via endocytosis acquire elevated SUMOylated hnRNPA1, stabilizing *PROX1* mRNA to increase PROX1 expression and lymphangiogenesis.

SUMOylation, small ubiquitin-like modifier binding, is an essential PTM mechanism that mediates protein stability and subcellular localization (11). SUMOylation is an essential process for packaging protein cargos into EVs. It was previously shown that hnRNPA1 is prone to SUMOylation (12) and that SUMOylated hnRNPA1 binds with small non-coding microRNAs to enhance their loading into EVs (13). Going deeper into understanding the hnRNPA1 modification by SUMOylation, Luo, Li, et al. showed that SUMOylation was triggered by the E1 SUMO-activating enzyme (SAE1), which depended on the activation of KRAS^G12D^ mutation-induced KRAS/RAF signaling. Experimental upregulation of SAE1 induced hnRNPA1 SUMOylation at lysine residue 113 (K113) (9). Further, SUMOylated hnRNPA1 K113 interacted directly with the tumor susceptibility gene 101 protein (TSG101), which increased the loading of SUMOylated hnRNPA1 into EVs (9). TSG101 is a key element for the endosomal sorting complex responsible for transport (ESCRT) mechanism. This component triggers EV synthesis and the development of intracellular vesicles that form multivesicular bodies. Subsequent fusion with the plasma membrane releases the vesicular cargo into the extracellular space (14). The interaction between TSG101 and SUMOylated hnRNPA1 K113 was essential for effective loading of the hnRNPA1 into PDAC EVs and lymphangiogenesis promotion (Figure 1) (9).

## From mutated KRAS to lymphangiogenesis

Luo, Li, et al. showed that HLECs from the local tumor microenvironment internalized EVs rich in SUMOylated hnRNPA1 that were released from PDAC cells by exocytosis (Figure 1) (9). Cytosolic PDAC-derived SUMOylated hnRNPA1 present in HLEC cells promoted tube formation and migration. The cytosolic hnRNPA1 promoted lymphangiogenesis in HLEC by upregulating PROX1 expression. PROX1 is a master regulator of lymphatic system development, necessary for lymphangiogenesis and essential for endothelial cell differentiation toward an HLEC phenotype (15).

Luo, Li, et al. showed that hnRNPA1 derived from PDAC EVs — once endocytosed and released into the cytosol — interacts with an AU-rich region in the *PROX1* 3′-untranslated region, increasing PROX1 expression by stabilizing the mRNA to increase its half-life. The hnRNPA1-induced lymphangiogenesis in HLEC cells takes place independent of VEGF-C, as it was shown that anti-VEGF-C antibodies did not interfere with the hnRNPA1 effect on PROX1 (9). The effect of KRAS^G12D^ PDAC EVs on LN metastasis and lymphangiogenesis was validated in a well-known model for PDAC KRAS^G12D^ (Kras^G12D/+^; Trp53^R172H/+^; Pdx-1-Cre (KPC) mice) (16). EVs with SUMOylated hnRNPA1 promoted LN metastasis in KPC mice, upregulated PROX1 expression, and promoted the development of the microlymphatic vessel network (9).

The clinical relevance of the in vitro and in vivo results was assessed using clinical samples from two PDAC KRAS^G12D^ patient cohorts. Overexpression of hnRNPA1 was observed in serum EVs isolated from PDAC patients when compared with healthy controls. This overexpression was associated with increased SAE1 and PROX1 expression and increased microlymphatic vessels. Importantly, hnRNPA1 performed better diagnostically at (a) identifying KRAS^G12D^ PDAC and (b) distinguishing between LN-positive and -negative disease, compared with classic tumor biomarkers carcinoembryonic antigen CEA0 and carbohydrate antigens CA19-9 and CA72-4.

## Conclusions and future directions

The insights into the mechanism of KRAS^G12D^ promotion of lymphangiogenesis in PDAC illustrated by Luo, Li, and colleagues open alternative research avenues for clinical application (9). Currently, identifying tumor spread to the LNs is realized using imaging methods, such as echoendoscopy, which are inaccurate in the early stages of pancreatic cancer (17). There is potential for hnRNPA1 to be further validated as a biomarker for screening for positive LN disease in KRAS^G12D^ PDAC. Additionally, the authors revealed a mechanism of lymphangiogenesis that uses an alternative pathway via PROX1 in a VEGF-C–independent manner (9). This finding offers important insights, as 30% of patients with PDAC with LN metastasis are unresponsive to targeted anti-VEGF-C therapy (18). A therapeutic approach should be envisioned that targets hnRNPA1 directly or targets regulatory elements involved in hnRNPA1 loading into EVs, such as KRAS^G12D^, SAE1, SUMO2 or TSG101. It would be of great interest to use the mechanism described by Luo, Li, et al. to check for KRAS-induced lymphangiogenesis in pancreatic cancers harboring other frequent KRAS mutations, such as G12C, G12V, or G12R. If the mechanism stands in multiple *KRAS*-mutated pancreatic cancers, exploring KRAS inhibitors may provide an elegant blocking strategy. In 2021, two molecules, sorafenib and adagrasib, were FDA approved for tumors harboring the *KRAS^G12C^* alteration, opening the avenue for KRAS-targeted therapies (19, 20). Also, other KRAS inhibitors undergoing preclinical investigations show promising results, moving the field toward early-phase clinical trials. Additional strategies could analyze approaches that combine classic anti-VEGF molecules with those targeting this VEGF-independent lymphangiogenesis pathway to better control *KRAS*-mutated pancreatic cancers. Targeting lymphangiogenesis has been a challenge in recent years and there are currently no FDA-approved molecules that specifically inhibit lymphangiogenesis (21). Previous approaches — focused on lymphangiogenesis-pathway regulators, such as VEGF-C and VEGFR-3 — had mixed results for both the in vitro and in vivo models (22). Therefore, a possible combination of VEGF-C, KRAS and hnRNPA1 inhibitors could enhance an inhibitory effect on the tumor-induced lymphangiogenesis.

In conclusion, Luo, Li, et al. unraveled a molecular mechanism behind tumor-induced lymph vessel development and early-LN metastasis in KRAS^G12D^ PDAC. The authors identified KRAS^G12D^ protein as an essential factor for VEGF-C-independent induction of lymphangiogenesis. Mechanistically, KRAS^G12D^ had a role in activating SAE1-dependent SUMOylation of hnRNPA1 with subsequent EV loading, which further induced PROX1 activation in HLEC (9) (Figure 1). This exciting basic research has substantial translational implications.

## Version 1 07/15/2022

Electronic publication
